# A Case of Esophageal Schwannoma and Review of the Literature

**DOI:** 10.7759/cureus.60109

**Published:** 2024-05-11

**Authors:** Zhongwei Wang

**Affiliations:** 1 Department of Radiology, Baoshan People's Hospital, Baoshan, CHN

**Keywords:** esophageal schwannoma, upper gastrointestinal radiography, mri, ct, esophagus, schwannoma

## Abstract

Malignant tumors are predominant in the esophagus, in which benign tumors are rare, and esophageal schwannoma is extremely rare. Here, we present a case of a 68-year-old woman with an unexpected chest computed tomography of inhomogeneous, well-defined, progressive delayed enhancement mass, and a paraesophageal lymph node. Mediastinal magnetic resonance imaging revealed an external growth mass in the lower esophagus, with an inhomogeneous signal, multiple internal cysts, and limited diffusion on diffuse-weighted imaging. Upper gastrointestinal radiography revealed a filling defect in the lower segment of the esophagus with no damage to the mucosal surface. Surgical resection and further pathological histology and immunohistochemical examination confirmed the diagnosis of esophageal schwannoma.

## Introduction

Schwannoma originates from Schwann cells of nerve fibers and is found frequently in intracranial nerve roots, spinal nerve roots, and peripheral nerves. Esophageal schwannoma originates from the esophageal plexus cell, with the most common being the vagus nerve [1]. It tends to occur in the upper and middle esophagus, and usually refers to a benign, slow-growing tumor, and has no specificity in clinical manifestations. Schwannomas of the gastrointestinal tract are rare, accounting for about 0%-3.4% of benign esophageal tumors, mainly in the stomach, followed by the rectum and colon, and rarely in the esophagus and small intestine [2]. Preoperative diagnosis is thought to be a challenge. A combined diagnosis by upper gastrointestinal radiography (UGI), CT, and MRI helps to improve the qualitative diagnostic ability [3]. Esophageal schwannoma is usually benign with a good prognosis. There is no report of tumor recurrence and metastasis after operation [4]. In this case, we present our UGI, CT, and MRI experience of an elder woman with no symptoms who was subsequently diagnosed with schwannoma by pathological results after surgical resection.

## Case presentation

A 68-year-old woman accidentally revealed a mediastinal mass after CT of the chest for one week. Two weeks ago, the patient was admitted to the outpatient department of another hospital for “intra-ear herpes pain.” During the course of the disease, the patient occasionally felt nausea and belching. A chest CT scan revealed a mass in the right lower mediastinum. The patient sought surgical treatment at our hospital.

An appendectomy and a left eye cataract surgery were performed before. She had previous chest trauma causing a rib fracture. The patient denied a history of diabetes, hypertension, blood transfusion, and other operations and trauma.

Imaging examination

**Figure 1:**
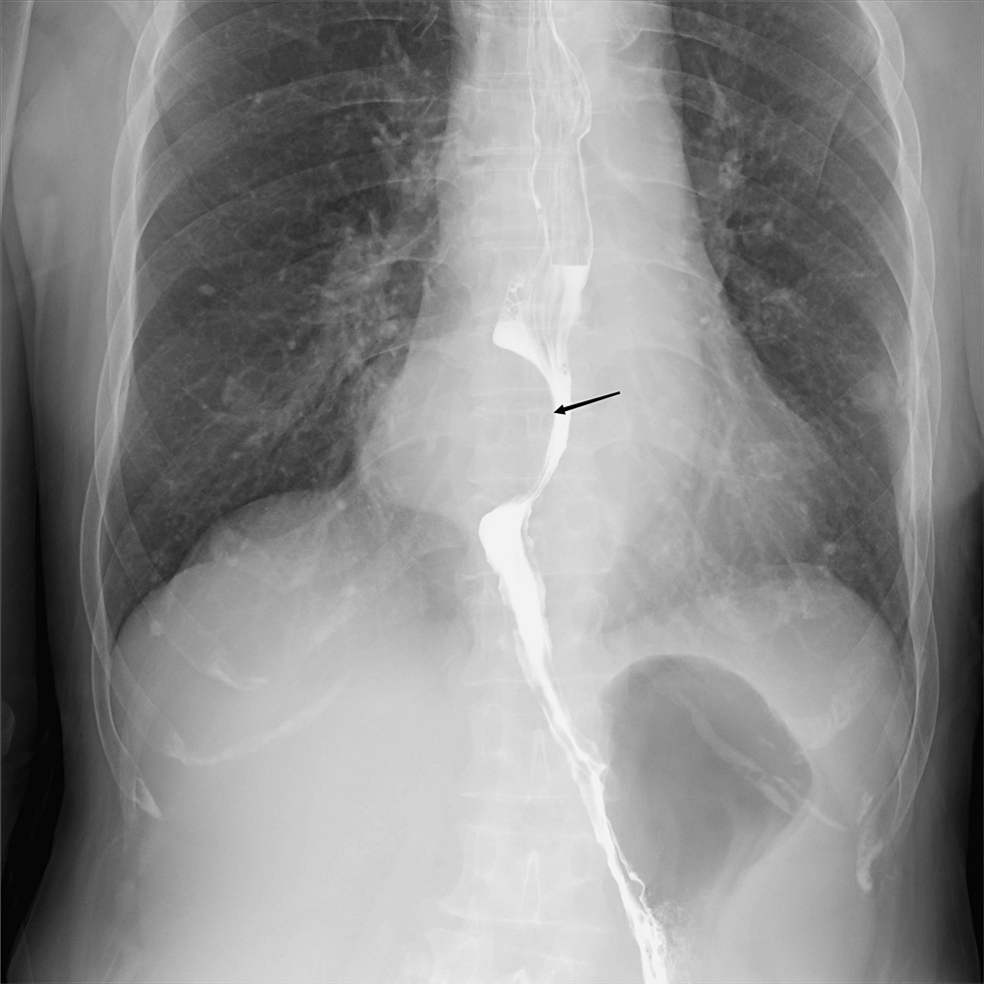
Upper gastrointestinal angiography

**Figure 2:**
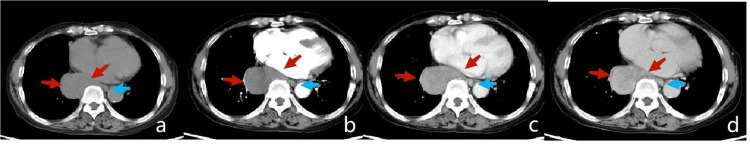
Plain CT and enhanced scan of the chest

**Figure 3:**
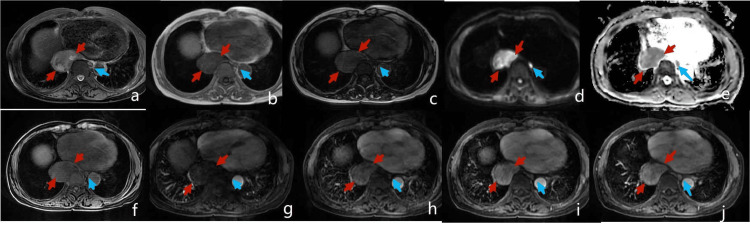
MRI of the mediastinum (a) T2-weighted imaging (T2WI). (b-c) T1-weighted imaging (T1WI) in and out of phase. (d-e) Diffusion-weighted imaging (DWI) and apparent diffusion coefficient (ADC) image. (f-j) T1WI of pre-enhancement. (f) Dynamic enhancement. (g-j) delayed enhancement.

UGI (Figure 1) showed a lower esophageal filling defect, with no obvious mucosal surface damage. A CT of the chest (Figure 2) showed a 4.0 × 5.4 cm, well-demarcated, inhomogeneous in density, moderate inhomogeneous progressive enhancement. Compared with adjacent muscles, MRI (Figure 3) showed a slightly lower signal on T1-weighted imaging, a slightly higher signal on T2-weighted imaging with scattered significantly high signal, limited diffusion on diffusion-weighted imaging (DWI), and moderate inhomogeneous progressive enhancement. A nodule on the left of the esophagus with a clear boundary, regular morphology, obvious diffusion limited, and homogenous enhancement suggested an enlarged lymph node.

Surgery and pathology

Video-assisted thoracoscopic surgery (VATS) with enucleation was performed to treat the lower esophageal tumor. Intraoperative freezing revealed a benign mesenchymal tumor. To reduce the intraoperative injury and improve the prognosis, no esophagectomy and lymph node dissection were performed.

**Figure 4:**
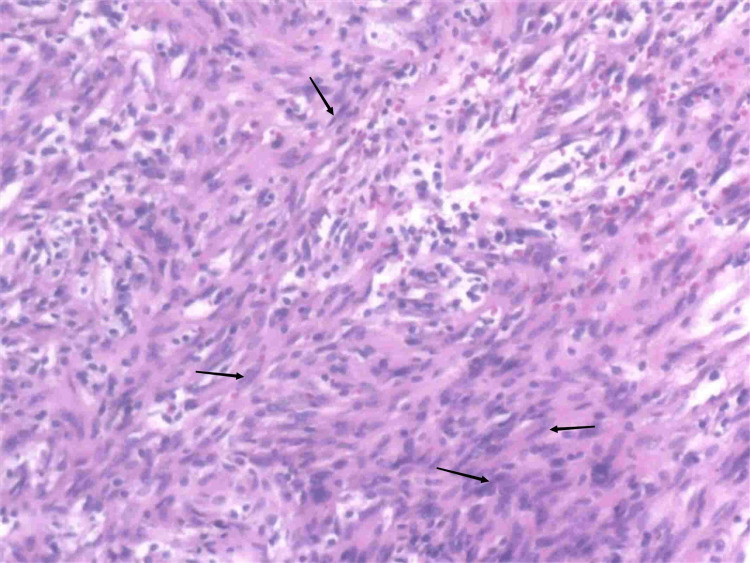
Histopathology image

The tumor cells are spindle-shaped and poorly defined between the cells. Some of the tumor cells were arranged in a palisade or incomplete vortex. Immunohistochemistry results were as follows: S-100 (+), SOX-10 (+), glial fibrillary acidic protein (GFAP) (foci +), vimentin (+), succinate dehydrogenase complex B (SDHB, +), CD99 (+), β catenin (+), smooth muscle actin (SMA, -), CD34 (vascular +), and CD117 (-), with a diagnosis of schwannoma (Figure 4).

One week after the operation, the patient's condition was stable and she was discharged.

## Discussion

Schwannomas are tumors derived from nerve sheath cells, which rarely occur in the gastrointestinal tract, and esophageal schwannomas are rare. Esophageal schwannoma has no obvious specific symptoms, which can cause dysphagia and dyspnea due to enlargement of the tumor and compression of adjacent structures. The degree of dysphagia has no direct correlation with the size of the tumor, it is related to the growth pattern of the tumor in or out of the esophageal lumen [5]. A retrospective study of 21 patients with surgically confirmed esophageal schwannoma showed most esophageal schwannomas appear to be homogeneously enhanced with infrequent cystic changes on CT and MRI in accordance with the features of predominant Antoni A patterns on pathological exam [3]. In this case, the tumor was large, transverse, and with exophytic growth, located in the lower esophagus, so there were no obvious symptoms of tumor compression with occasional nausea and belch. Esophageal schwannoma occurs more frequently in the upper esophagus than in the lower esophagus. This case was located in the lower esophagus with a filling defect in the UGI and no destruction of the mucosal surface, suggesting the origin of the submucosal or muscle layer and exophytic growing, consistent with the literature [3]. The density of the tumor on plain CT was slightly inhomogeneous. MRI T2-weighted imaging (T2WI) could clearly show a slightly higher signal with the scattered high signal area. T2WI high signal area had a mild delay enhancement, which reflected the pathological bundle Antoni A rich cells, internal scattered mucus edema sample sparse network Antoni B area, and lack of obvious cystic necrosis, which indicated that MRI can better reflect the tumor‘s pathology. The inhomogeneous density and signal intensity in this case may be related to the larger tumor volume. Scattered lymphocytes and nodular lymphatic sleeves scattered in tumors were generally considered the characteristic morphology of schwannoma in the digestive tract [3]. While esophageal schwannomas are benign tumors, they show a hypermetabolic appearance on 18-fluorodeoxyglucose positron emission tomography (PET), similar to findings of malignant esophageal tumors [6]. PET was performed in two cases of esophageal schwannoma, and both of them showed fluorodeoxyglucose (FDG) accumulation and mediastinal lymph node uptake. All the lymph nodes with FDG were proved to be reactive hyperplasia by postoperative pathology [3]. Perilesional lymph nodes were important signs distinguishing gastric schwannoma from gastric stromal tumor [7]. Similarly, esophageal tumors with benign characteristics and peritumoral lymph nodes may improve confidence in the diagnosis of schwannoma.

The imaging differential diagnosis of esophageal schwannoma mainly includes leiomyoma and stromal tumor. Esophageal leiomyoma is deformed along the smooth muscle, usually longer than the diameter of the spindle or irregular shape, with mild enhancement, and the degree of enhancement is lower than that of schwannoma. Stromal tumors may have intraluminal, extra-luminal, and mixed growth patterns, but the enhancement was markedly inhomogeneous, and the degree was higher than that in schwannomas, which was significantly different from that in schwannomas [3,8]. Finally, by immunohistochemistry, esophageal schwannoma was positive for S-100, esophageal leiomyoma was positive for SMA, and esophageal stromal tumor was positive for CD34 and CD117 [3].

At present, surgical resection is an effective method for the treatment of esophageal schwannoma, and the prognosis is good. With the development and application of VATS, more and more benign esophageal tumors are resected by VATS. Esophageal schwannoma is usually benign with a good prognosis. There is no report of tumor recurrence and metastasis after operation [4]. In this case, despite the large size of the tumor, VATS was performed and the patient recovered well.

## Conclusions

Esophageal schwannoma is rare. Preoperative diagnosis could be made by UGI, CT, and MRI, through round or oval morphology, exophytic growth, lateral growth, T2WI bipolar characteristics, and moderate inhomogeneous progressive enhancement. In esophageal tumors with benign imaging features, the appearance of paralesional lymph nodes may be a characteristic performance.
